# Autophagy is involved in the toxicity of the biocontrol agent GC16 against *Tetranychus pueraricola* (Acari: Tetranychidae) based on transcriptomic and proteomic analyses

**DOI:** 10.1186/s12864-025-11312-7

**Published:** 2025-02-07

**Authors:** Yanyan He, Guangzu Du, Guang Wang, Huiming Guan, Shusheng Zhu, Bin Chen, Xiahong He, Youyong Zhu

**Affiliations:** 1 School of Agriculture, Yunnan University, Kunming, 650500 China; 2 State Key Laboratory of Conservation and Utilization of Biological Resources of Yunnan, College of Plant Protection, Yunnan Agricultural University, Kunming, 650201 China; 3 Southwest Forestry University, Kunming, 650224 China; 4 Inner Mongolia Wulanchabu Science and Technology Development Center, Wulanchabu, 012000 China; 5 Yunnan Provincial Center for Disease Control and Prevention, 650034 Kunming, China

**Keywords:** GC16 biopesticide, Autophagy, Transcriptomic, Proteomic, Toxicity, *Tetranychus Pueraricola*

## Abstract

**Background:**

GC16 is a novel pesticide with acaricidal properties against the spider mite *Tetranychus pueraricola* (Ehara & Gotoh). Its physiological mechanisms have been described previously, but its molecular mechanisms of action remain unclear. Thus, we aimed to explore the acaricidal mechanisms of GC16 through transcriptomic and proteomic analyses. The results were verified using transmission electron microscopy (TEM), immunofluorescence assay, and western blotting.

**Results:**

Transcriptomic and proteomic analyses revealed 2717 differentially expressed genes (DEGs) and 374 differentially expressed proteins (DEPs) between the GC16-treated and control mites. Kyoto Encyclopedia of Genes and Genomes (KEGG) enrichment analysis indicated that the DEGs and DEPs were enriched in the autophagy pathway. TEM showed that the number of autophagosomes and autolysosomes was higher in the GC16-treated mites than in the control mites. Immunofluorescence assay and western blot results consistently indicated that GC16 treatment significantly enhanced the relative expression of the autophagy protein LC3 in insect Sf9 cells. The intracellular calcium concentration in the GC16-treated Sf9 cells was 2.30 times higher than that in the control cells, suggesting that GC16 disrupted calcium homeostasis and potentially acted as a calcium-driven nerve agent.

**Conclusions:**

Autophagy is involved in the toxicity of GC16 against *T. pueraricola* and may be activated by elevated Ca^2+^ levels. This study reveals the molecular insecticidal mechanisms of GC16 and provides rationale for the field application of GC16 to control pest mites.

**Supplementary Information:**

The online version contains supplementary material available at 10.1186/s12864-025-11312-7.

## Background

The spider mite *Tetranychus pueraricola* (Ehara & Gotoh; Acari: Tetranychidae) is an important agricultural pest mite [1–2]. Phylogenetically and morphologically, *T. pueraricola* is closely related to *Tetranychus urticae* Koch (red form) [3]. It can be distinguished from *T. urticae* based on the size of the knob on the aedeagus, which is approximately 2.1 μm in *T. pueraricola* and approximately 2.5 μm in *T. urticae* [4]. *T. pueraricola* is widely distributed in China and Japan and has caused significant economic losses to local agriculture, especially in southwest China [4–5]. In addition, *T. pueraricola* is a key factor restricting the growth of Chinese herb *Panax notoginseng* (Burk.) F. H. Chen and *Polygonatum kingianum* Coll. et Hemsl, according to field surveys [2, 6]. Despite the promotion of integrated pest management, chemical control remains a prominent method for managing *Tetranychus* mites owing to its rapid effects. However, the long-term, extensive use of conventional acaricides has resulted in the development of acaricide resistance in spider mites [7–8]. Furthermore, pesticide residue represents a major threat to both agricultural ecosystems and human health [9]. The development of new ecological acaricides is a crucial step toward the advancement of sustainable agriculture as alternatives to synthetic chemical acaricides.

GC16 is a novel eco-friendly pesticide composed of 55% lecithin (soybean extract) and 45% calcium chloride (CaCl_2_). This compound has been reported to be effective in controlling spider mites, thrips, and whiteflies [10]. GC16 has yet to be officially registered and is currently undergoing field trials across 15 provinces in China [2]. We previously explored the acaricidal activities and physiological mechanisms of GC16 against *T. pueraricola* [6] and evaluated its sublethal effects on the development and enzyme activities of this mite species [2]. However, the molecular mechanisms underlying the acaricidal activity of GC16 against *T. pueraricola* remain elusive.

Transcriptome and proteome sequencing are frequently employed to analyze the molecular mechanisms of pesticides [11–12]. Transcriptome sequencing is a valuable tool for identifying key genes associated with acaricidal activity [13–14]. Similarly, comparative proteomic analysis can be employed to ascertain the pivotal metabolic pathways related to pesticide toxicity [15–17].

The mechanism of action of pesticides may involve the alteration of metabolic pathways. Additionally, some studies have indicated a correlation between the toxicity of pesticides and autophagy in insects [18–19]. Autophagy is a self-cannibalization process triggered by stress signals that maintains cellular health by efficiently replacing dysfunctional cellular components with new components [20–21]. This process plays a fundamental role in the growth, development, and homeostasis of eukaryotic cells [22–24], including insect cells [25]. Transmission electron microscopy (TEM), immunofluorescence assay, and western blotting are widely used in autophagy studies [26–27].

Studies on the action mechanisms of acaricides typically highlight the importance of calcium homeostasis [28–31]. Calcium ions (Ca^2+^) are crucial secondary messengers in cellular signaling, and maintaining intracellular Ca^2+^ homeostasis is fundamental to various biological activities [18, 32]. Elevated intracellular free calcium ion ([Ca^2+^]i) levels are associated with the neurotoxic properties of rotenone [33]. Furthermore, a complex regulatory network exists between Ca^2+^ signaling and autophagy [34–36]. Beclin 1 is a crucial element in the primary generation of autophagosomes, and it has been suggested to intersect with numerous Ca^2+^ signaling pathways [37]. Increased Ca^2+^ levels in the abnormal silk gland of *Bombyx mori* after exposure to chlorantraniliprole (CAP) promote autophagy, resulting in decreased energy metabolism and insufficient ATP synthesis in the mitochondria [18]. However, the roles of autophagy and Ca^2+^ in the acaricidal activity of GC16 remain unclear.

This study aimed to elucidate the acaricidal mechanisms of GC16 against *T. pueraricola.* RNA-seq and label-free quantitative LC/MS proteomics techniques were used to identify DEGs and DEPs in *T. pueraricola* under different treatments. Key signaling pathways were enriched at the transcriptomic and proteomic levels. The findings were verified using TEM, immunofluorescence assay, and western blotting, and the [Ca^2+^]i levels in insect Sf9 cells were measured. The results of this study may serve as a basis for applying GC16 to control spider mites and elucidating the mechanisms of action of new pesticides.

## Methods

### Spider mite rearing and sample preparation

The spider mite *T. pueraricola* was collected in 2021 from a Chinese herb planting area in Lancang County, Yunnan, China, and maintained on insect-free and pesticide-free kidney bean plants in a greenhouse at Yunnan Agricultural University, Kunming, Yunnan Province, China, under the following conditions: 25 ± 2 °C, 65 ± 5% RH, and light: dark photoperiod of 16:8 h.

GC16 composed of 45% CaCl_2_ and 55% lecithin was prepared as described previously [2]. For transcriptomic and proteomic sequencing, a total of approximately 800 fresh, healthy, and uniform female mites were selected from the mite population for each replicate (triplicate for each treatment). These mites were then placed on new bean plants. Subsequently, these plants were evenly sprayed with GC16 (2.10 mg/mL, LC_50_), CaCl_2_ (0.95 mg/mL), lecithin (1.15 mg/mL), or water (control) using a power sprayer. Subsequently, the plants were transferred to separate insect-rearing cages (200 mesh, 50 cm × 50 cm × 50 cm) within a climate chamber set to a temperature of 26 ± 2 °C, relative humidity of 65 ± 5%, and light: dark photoperiod of 14:10 h. After 12 h, the surviving individuals were collected with the aid of a small brush in aseptic, enzyme-free centrifuge tubes of 1.5 mL. The tubes were then immediately frozen in liquid nitrogen and stored at − 80 ℃.

Total RNA was extracted using an animal tissue total RNA extraction kit (Tiangen Biochemical Technology Co., Ltd., Beijing, China) in accordance with the manufacturer’s instructions. RNA degradation was monitored on 1% agarose gels, and its purity was assessed using a NanoPhotometer spectrophotometer (IMPLEN, CA, USA). RNA concentration was measured using the Qubit^®^ RNA Assay Kit (Life Technologies, CA, USA). RNA integrity was assessed using the Agilent Bioanalyzer 2100 system (Agilent Technologies, CA, USA).

### Transcriptome sequencing and differential expression analysis

In accordance with the methodology described by He et al. [38], transcriptome sequencing was performed by Novogene Co., Ltd. (Beijing, China) on an Illumina Novaseq platform, generating 150 bp paired-end reads. Raw data (raw reads) in fastq format were first processed using fastp. Clean reads were obtained by removing low-quality reads and reads containing adapter and 1 poly-N. As *T. pueraricola* genome has not yet been sequenced, the *T. urticae* genome was employed as a reference owing to the close evolutionary relationship between the two species. Reference genome files were downloaded from the NCBI database (version: ensemblmetazoa_tetranychus_urticae_asm23943v1_gca_000239435_1) [39]. A reference genome index was established using Hisat2 v2.0.5, and clean paired-end reads were aligned with the reference genome using Hisat2 v2.0.5. The mapped reads of each sample were assembled using StringTie (v1.3.3b) [40]. FeatureCounts v1.5.0-p3 was used to count the number of reads mapped to each gene. Unigene expression was represented in fragments per kilobase of transcript per million mapped reads (FPKM) value [41]. DEGs were screened using DESeq setting with adjusted p-value ≤ 0.05 and|fold change| ≥ 1. Functional annotation and enrichment analysis were performed using Gene Ontology (GO), and metabolic pathway analysis was performed using Kyoto Encyclopedia of Genes and Genomes (KEGG) with the ClusterProfiler R package, in which gene length bias was corrected, and the species was selected as *T. urticae*. GO terms with corrected p-values < 0.05 were considered significantly enriched by DEGs.

### Protein extraction, trypsin digestion, and UHPLC-MS/MS analyses

Label-free quantitative LC/MS proteomics was employed for proteomic sequencing [42], which was performed by Novogene Co., Ltd. (Beijing, China). Total protein was extracted as previously described by Winiewski et al. [43] and Wu et al. [44]. Samples were ground and lysed with SDT lysis buffer (containing 100 mM NaCl) and 1/100 volume of DL-dithiothreitol. Protein concentration was determined using a Bradford protein quantification kit (Beyotime Biotechnology, Shanghai, China). Each protein sample was digested with trypsin and loaded onto a C_18_ desalting column, followed by lyophilization for further separation of fractions [45].

UHPLC-MS/MS analyses were performed using an EASY-nLC™ 1200 UHPLC system (Thermo Fisher Scientific, Germany) coupled with a Q Exactive HF-X mass spectrometer (Thermo Fisher Scientific). The resulting spectra were subjected to a search against the 1,962,301.fasta database (comprising 15684 sequences), downloaded from the NCBI website [39] and designated as GCF_000239435.1_ASM23943v1_protein.faa. The search was conducted using the Proteome Discoverer 2.5 search engine (Thermo, HFX, and 480). Protein quantitation results were statistically analyzed using *t*-test. Proteins whose quantitation results were significantly different between the experimental and control groups (*p* < 0.05,|fold change| > 1.5) were considered DEPs. GO and InterPro (IPR) functional analyses were conducted using the InterProScan program against non-redundant protein databases (including Pfam, PRINTS, ProDom, SMART, ProSite, and PANTHER) [46], and KEGG databases were used to analyze the protein families and pathways. DEPs were used for GO, IPR, and KEGG enrichment analyses [47].

### TEM observation

Autophagosomes and autolysosomes in mites were observed via TEM. A total of 100 fresh, healthy, and uniform female mites were selected from the mite population for each treatment. The mites were then transferred to new bean plants. These plants were evenly sprayed with GC16 (2.10 mg/mL, LC_50_), CaCl_2_ (0.95 mg/mL), lecithin (1.15 mg/mL), or water (control), using a power sprayer. Subsequently, the plants were transferred to separate insect-rearing cages (200 mesh, 50 cm × 50 cm × 50 cm) within a climate chamber set to a temperature of 26 ± 2 °C, relative humidity of 65 ± 5%, and L: D photoperiod of 14:10 h. After 24 h, the surviving mites were collected with the aid of a small brush for subsequent TEM observation. In accordance with our previously described method [6], mite samples were fixed with 2.5% glutaraldehyde in 0.1 M phosphate buffer (PB, pH 7.4) and post-fixed with 1% OsO_4_, dehydrated in a series of ethanol, transitioned in acetone, embedded in Epon 812 resin, and then polymerized at 60 °C. Serial ultrathin sections with uniform thickness (60 nm) were prepared using a Leica EM UC7 ultramicrotome (Leica, Weztlar, Germany). Ultrathin sections were loaded onto 50-mesh Cu grids and double-stained with 2% uranyl acetate and lead citrate before observation under a JEM 1400 Plus transmission electron microscope (JEOL, Tokyo, Japan) at 120 kV.

### Immunofluorescence assay

Owing to the infeasibility of cultivating spider mite cells in vitro, insect Sf9 cells were used to detect the expression of autophagy protein LC3. Sf9 cells were cultured on coverslips and treated with water (control) or 2.1 mg/mL GC16 for 24 h. The cells were fixed with 4% paraformaldehyde for 15 min, permeabilized with 0.5% Triton X-100 at 25 °C for 5 min, and then blocked (AR1009; BOSTER Biological Technology Co., Ltd., Wuhan, China) at 25 °C for 30 min. LC3 expression was detected by incubating the samples with primary antibodies against LC3 (AP0762, Bioworld Technology, Inc., USA) at 4 °C overnight and then with corresponding secondary antibodies (BA1032, BOSTER Biological Technology Co. Ltd, Wuhan, China) at 37 °C for 1 h. Subsequently, the samples were stained with DAPI (C1002, Beyotime Biotechnology, Shanghai, China) for 5 min, and covered with a sealing agent to prevent fluorescence quenching (0100-01, Southern Biotechnology Associates, Inc., USA). The coverslips were allowed to dry, and then images were taken using a laser confocal microscope (FV3000, Olympus, Japan). Nuclei are depicted in blue, whereas the LC3 protein is indicated in red.

### Western blot analysis

LC3-I/II expression was quantified using western blotting in accordance with a previously described methodology [48]. Insect Sf9 cells were inoculated in cell culture plates and then treated with water (control) and 2.1 mg/mL GC16 for 24 h. Total protein was extracted after the treatment. Proteins were separated using 12% sodium dodecyl sulfate-polyacrylamide gel electrophoresis (SDS-PAGE). The PAGE gel was then transferred onto polyvinylidene membranes. After being blocked with TBST containing 5% skim milk powder for 2 h at room temperature, the membranes were incubated with primary antibodies against LC3 (1:2000, 14600-1-AP, Proteintech) and β-actin (1:20000, T0022, Affinity Biosciences) overnight at 4 °C. Subsequently, the membranes were incubated with corresponding HRP-conjugated secondary antibodies LC3 (1:10000, A0208, Beyotime Biotechnology) and β-actin (1:10000, SA00001-1, Proteintech) for 2 h at 25 °C. β-actin was used as the loading control to normalize the protein content. Protein expression was quantified using an enhanced chemiluminescence kit (G2014, Servicebio). The Image-Pro Plus software was used to analyze protein bands.

### Reactive oxygen species (ROS) level assay

Sf9 cells were continuously exposed to water (control) or 2.1 mg/mL GC16 for 30 min and 6, 12, and 24 h. Then, ROS levels were detected according to the instructions provided with the Reactive Oxygen Species Assay Kit (S0033S, Beyotime Biotechnology, Shanghai, China), using flow cytometry (CytoFLEX, BECKMAN COULTER Life Sciences, California, USA).

### Apoptosis assay

Sf9 cells were continuously exposed to water (control) or 2.1 mg/mL GC16 for 24 h. Then, apoptosis was detected according to the instructions provided with AnnexinV-FITC/PI double dye cell apoptosis detection kit (QS-S306, Keycell, Wuhan, China), using flow cytometry (CytoFLEX, BECKMAN COULTER Life Sciences, California, USA).

### [Ca2+]i assay

[Ca^2+^]i levels were assessed using Fluo-4/AM fluorescence staining [30]. Insect Sf9 cells were treated with water (control), 2.1 mg/mL GC16, 0.95 mg/mL CaCl_2_, or 1.15 mg/mL lecithin for 24 h. Finally, the intracellular [Ca^2+^]i levels were measured.

### Data analysis

Statistical analyses were performed using SPSS (version 25.0; SPSS Inc., Chicago, IL, USA), and differences were determined using Student’s *t*-test (for two treatments) or Tukey’s honestly significant difference test (for three or more treatments). Differences were considered statistically significant at *p* < 0.05.

## Results

### Transcriptome analysis

A total of 74.64 gigabytes of clean data were generated via transcriptome sequencing, with each sample producing over 6.12 gigabytes (Table S1). The percentage of bases with a quality value greater than Q30 exceeded 93.11%, confirming high-quality and sufficient sequencing data (Table S1). Furthermore, principal component analysis (PCA) results showed a substantial variance between the GC16 and control groups (Figs. S1 and S2).

In total, 15,400 genes were expressed, and 2717 DEGs (1338 upregulated and 1379 downregulated) were identified in the comparison of the GC16 and control groups. In addition, 2212 (1060 upregulated and 1152 downregulated) and 1729 (811 upregulated and 918 downregulated) DEGs were identified in the comparison of lecithin vs. control groups and CaCl_2_ vs. control, respectively (Table S2).

GO enrichment analysis revealed that the DEGs in the GC16 vs. control comparison were significantly enriched in (intracellular) non-membrane-bound organelles, ribonucleoprotein complexes, cellular amide, and peptide metabolic and biosynthetic processes, structural molecule activity, and ribosomes (Fig. S3). This result indicated that GC16 treatment affected the organelles, particularly the ribosomes, and the biosynthesis and metabolism of amide and peptide compounds in the mites.

Furthermore, the DEGs in the comparison of lecithin vs. control and CaCl_2_ vs. control were highly enriched in the (intracellular) non-membrane-bound organelles. The enrichment of DEGs in the lecithin vs. control comparison was similar to that of DEGs in the GC16 vs. control comparison, indicating that GC16 and lecithin had the potential to alter ribosomal structure and impact amide and peptide reactions (Fig. S3).

**Fig. 1:**
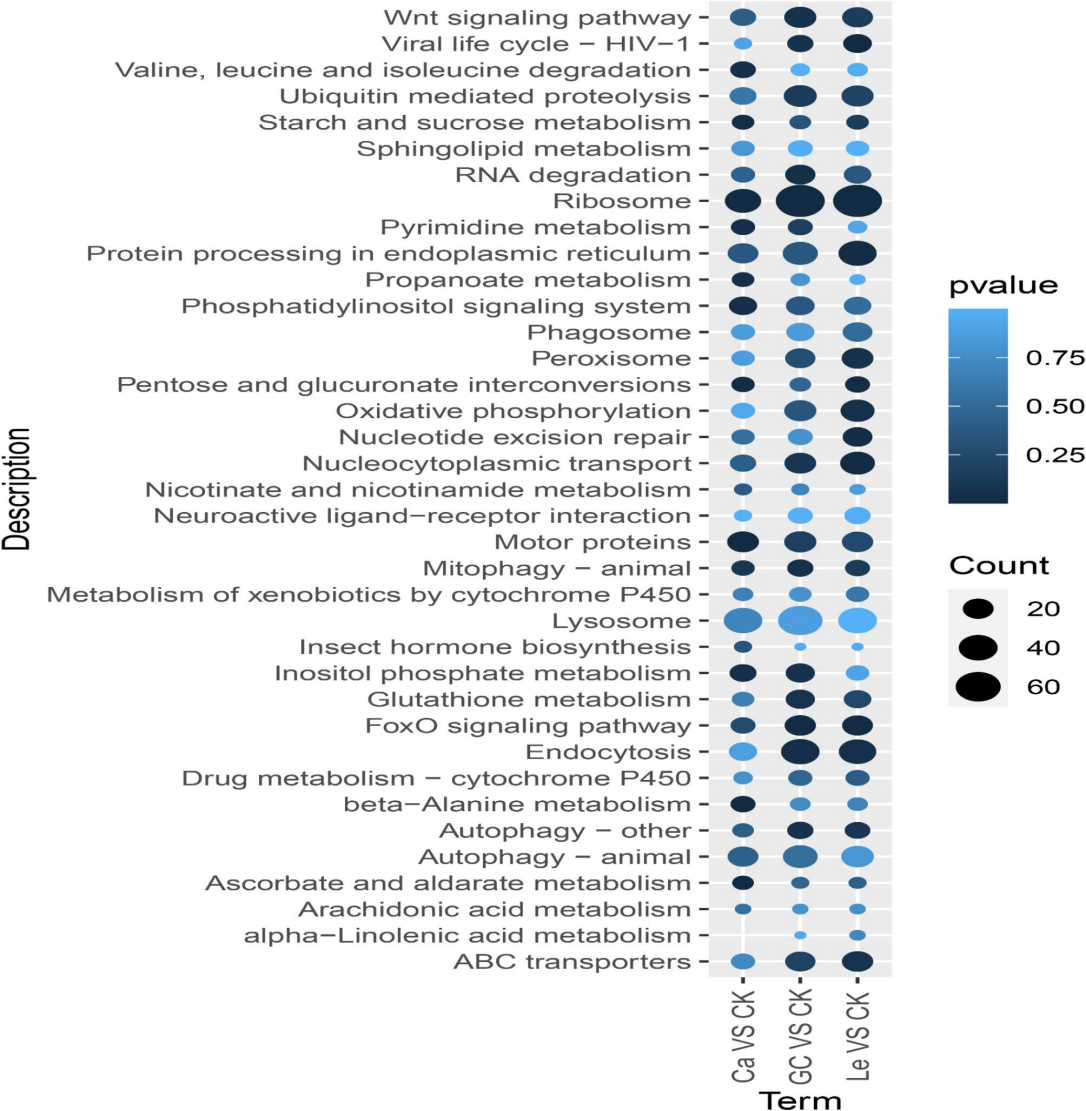
KEGG enrichment of the differentially expressed genes (DEGs) between GC16 (GC) and Control (CK), Lecithin (Le) and Control (CK), and CaCl_2_ (Ca) and Control (CK). The abscissa is the compared group of differential treatments, and the ordinate is KEGG pathway

Results of KEGG enrichment analysis showed that the DEGs in the GC16 vs. control comparison were significantly enriched in various pathways, including the ribosomes, endocytosis, ubiquitin-mediated proteolysis, Wnt signaling pathway, motor proteins, nucleocytoplasmic transport, FoxO signaling pathway, ABC transporters, peroxisomes, inositol phosphate metabolism, glutathione metabolism, mitophagy - animal, and autophagy - other (Fig. 1).

The DEGs in the lecithin vs. control comparison were enriched in key pathways, including the ribosomes, endocytosis, protein processing in the endoplasmic reticulum, oxidative phosphorylation, nucleocytoplasmic transport, mitophagy - animal, and autophagy - other (Fig. 1). Similarly, the DEGs in the CaCl_2_ vs. control comparison were enriched in the ribosomes, motor proteins, and phosphatidylinositol signaling systems (Fig. 1). These results indicated that all three treatments (GC16, lecithin, and CaCl_2_) significantly affected the ribosome pathway. GC16 and lecithin affected endocytosis, nucleocytoplasmic transport, mitophagy, and autophagy. Notably, lecithin affected oxidative phosphorylation, and CaCl_2_ regulated the phosphatidylinositol signaling system.

GO and KEGG enrichment results consistently suggested that GC16 treatment affected the ribosomes. KEGG analysis indicated that the autophagy pathway was affected by GC16, suggesting that autophagy was involved in the acaricidal activity of GC16.

### Proteomic analysis

In total, 3277 proteins were identified via proteomic sequencing (Table S3). PCA results indicated that the GC16 and control samples were segregated into two distinct groups. These findings indicated that GC16 was the principal determinant responsible for the observed differences in protein expression between the two groups (Figs. S4 and S5).

A total of 2882 expressed proteins and 374 DEPs (338 upregulated and 36 downregulated) were identified in the GC16 vs. control comparison. Additionally, 120 (105 upregulated and 15 downregulated) and 62 (36 upregulated and 26 downregulated) DEPs were identified in the comparison of lecithin vs. control and CaCl_2_ vs. control, respectively (Table S4).

GO enrichment analysis indicated that the DEPs in the GC16 vs. control comparison were significantly enriched in functions related to binding, organic cyclic compound binding, heterocyclic compound binding, nucleic acid binding, nucleoside triphosphatase activity, and ATPase activity (Fig. S6). The DEPs in the lecithin vs. control comparison were enriched in functions related to the binding of organic cyclic compounds, heterocyclic compounds, and nucleic acids (Fig. S6). The DEPs in the CaCl_2_ vs. control comparison were enriched in functions related to binding and vacuolar transport (Fig. S6). In conclusion, GC16 and lecithin affected the binding of organic cyclic compounds, heterocyclic compounds, and nucleic acids.

**Fig. 2:**
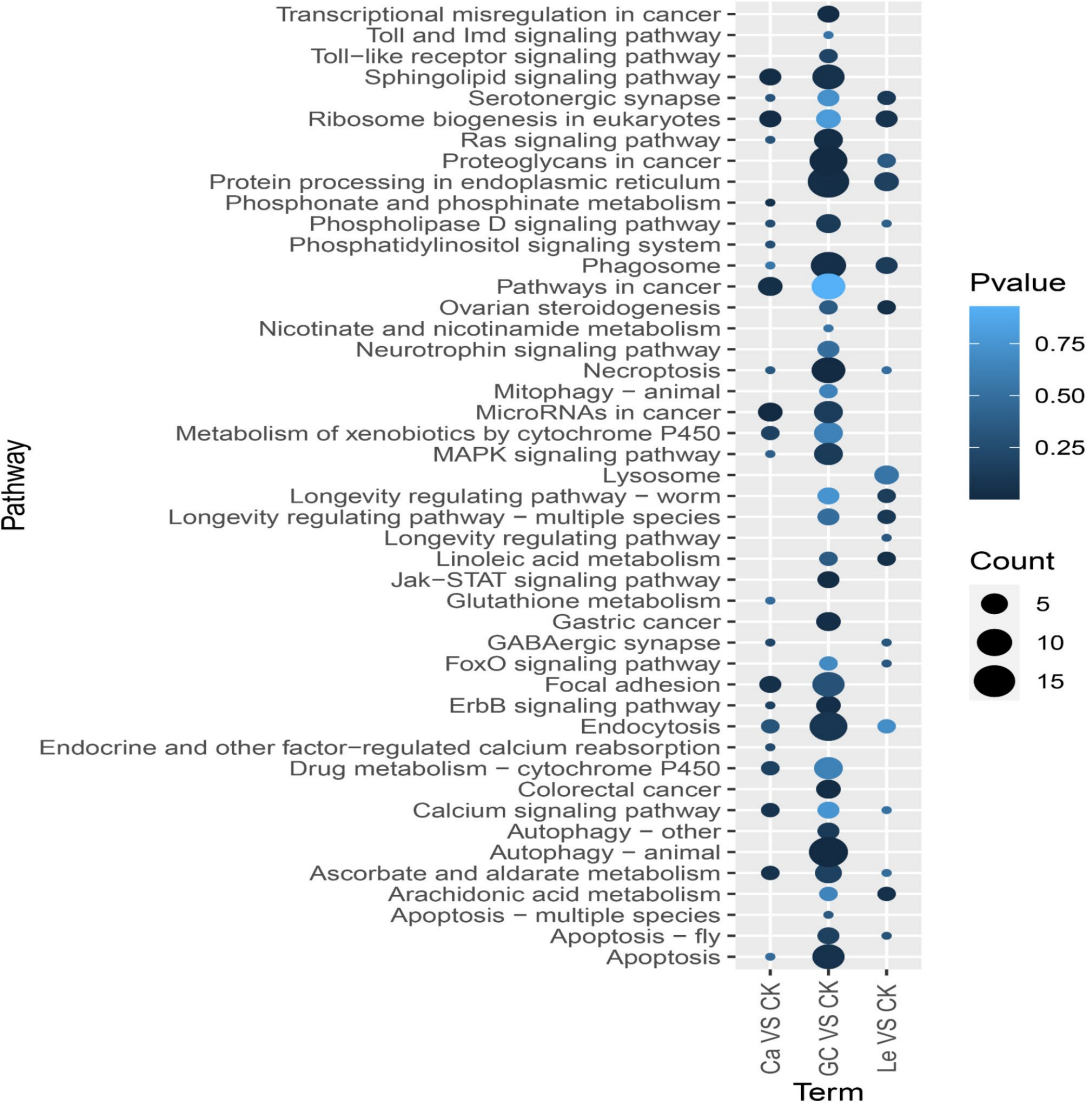
KEGG enrichment of the differentially expressed proteins (DEPs) between GC16 (GC) and Control (CK), Lecithin (Le) and Control (CK), and CaCl_2_ (Ca) and Control (CK). The abscissa is the compared group of differential treatments, and the ordinate is KEGG pathway

KEGG enrichment analysis revealed that the DEPs in the GC16 vs. control comparison were enriched in various pathways, including autophagy - animal, necroptosis, proteoglycans in cancer, protein processing in the endoplasmic reticulum, phagosome, apoptosis, and sphingolipid signaling pathway (Fig. 2). The DEPs in the lecithin vs. control comparison were enriched in protein processing in the endoplasmic reticulum, phagosomes, ribosome biogenesis in eukaryotes, arachidonic acid metabolism, linoleic acid metabolism, and ovarian steroidogenesis (Fig. 2). The DEPs in the CaCl_2_ vs. control comparison were enriched in microRNAs in cancer, pathways in cancer, focal adhesion, sphingolipid signaling pathway, ribosome biogenesis in eukaryotes, and calcium signaling pathway (Fig. 2).

These results indicated that GC16 affected autophagy and apoptosis in mites. Additionally, GC16 and lecithin influenced the endoplasmic reticulum and phagosomes, whereas GC16 and CaCl_2_ affected the sphingolipid signaling pathway. Furthermore, CaCl_2_ affected the calcium signaling pathway.

### Expression of DEGs and DEPs in key pathways

**Fig. 3:**
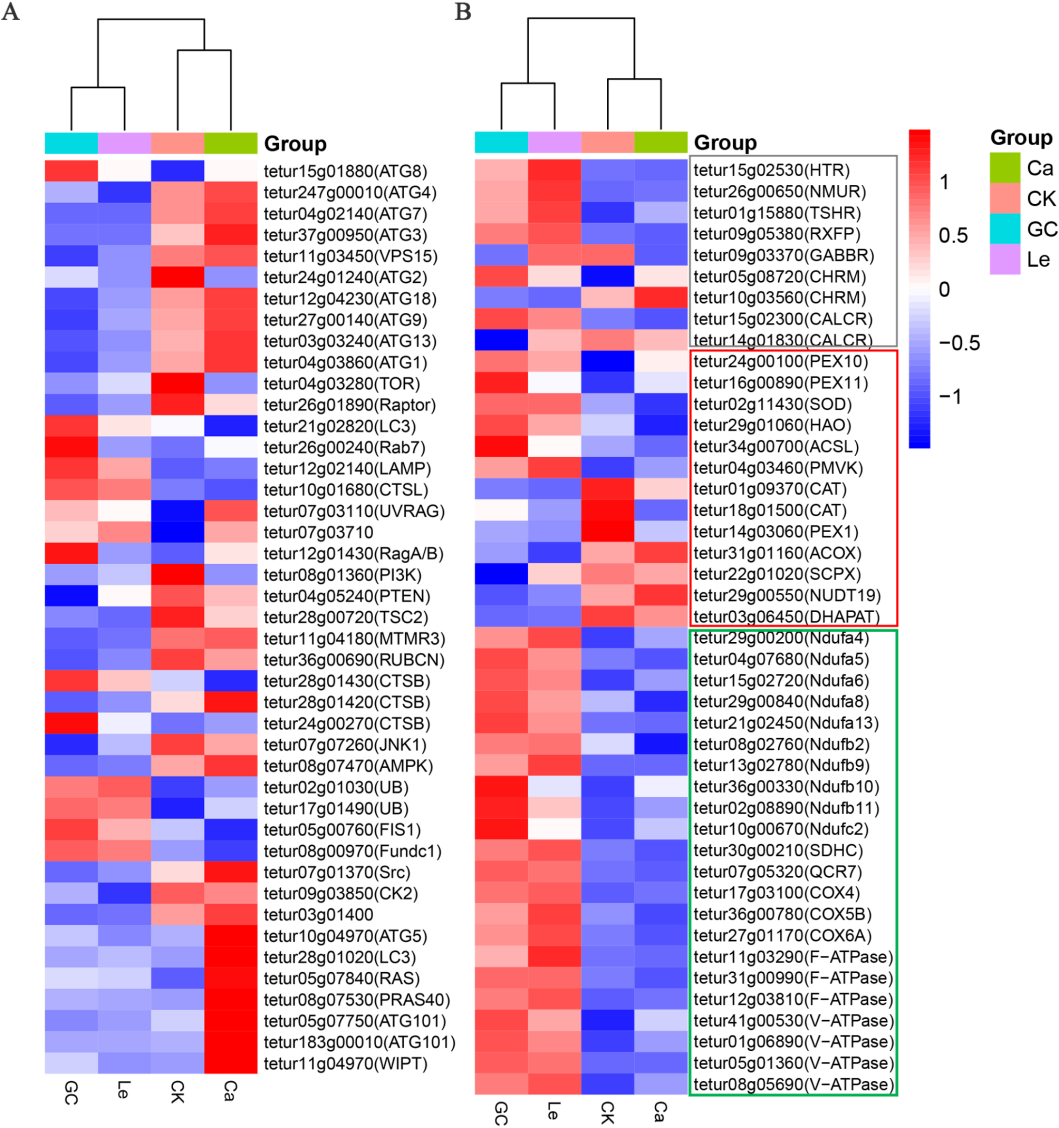
Heatmap of the differentially expressed genes (DEGs) enriched in key KEGG pathways. (**A**) Autophagy and Mitophagy pathway. (**B**) Oxidative phosphorylation (marked in green), Neuroactive ligand-receptor interaction (marked in grey), and Peroxisome (marked in red) pathway. GC16: GC; Control: CK; Lecithin: Le; CaCl_2_: Ca

The DEGs related to autophagy, mitophagy, oxidative phosphorylation, neuroactive ligand–receptor interactions, and peroxisome pathway were subjected to transcriptomic analysis (Fig. 3). GC16 treatment upregulated the expression of all DEGs involved in the oxidative phosphorylation pathway. In the autophagy pathway, GC16 treatment inhibited the expression of the ubiquitin-like-conjugating enzyme ATG3, ubiquitin-like modifier-activating enzyme ATG7, and gamma-aminobutyric acid receptor-associated protein ATG8 while increasing the expression of microtubule-associated proteins 1 A/1B light chain 3 A (tetur21g02820, LC3). Additionally, GC16 treatment downregulated the expression of gamma-aminobutyric acid type B receptor 1 in the neuroactive ligand–receptor interaction pathway as well as catalase and peroxisomal biogenesis factor 1 (PEX1) in the peroxisome pathway. However, GC16 treatment upregulated the expression of PEX10, PEX11, and superoxide dismutase in the peroxisome pathway. Transcriptomic analysis revealed that GC16 treatment disrupted the pathways of autophagy, mitophagy, and oxidative phosphorylation, indicating that it hindered cellular metabolic homeostasis.

**Fig. 4:**
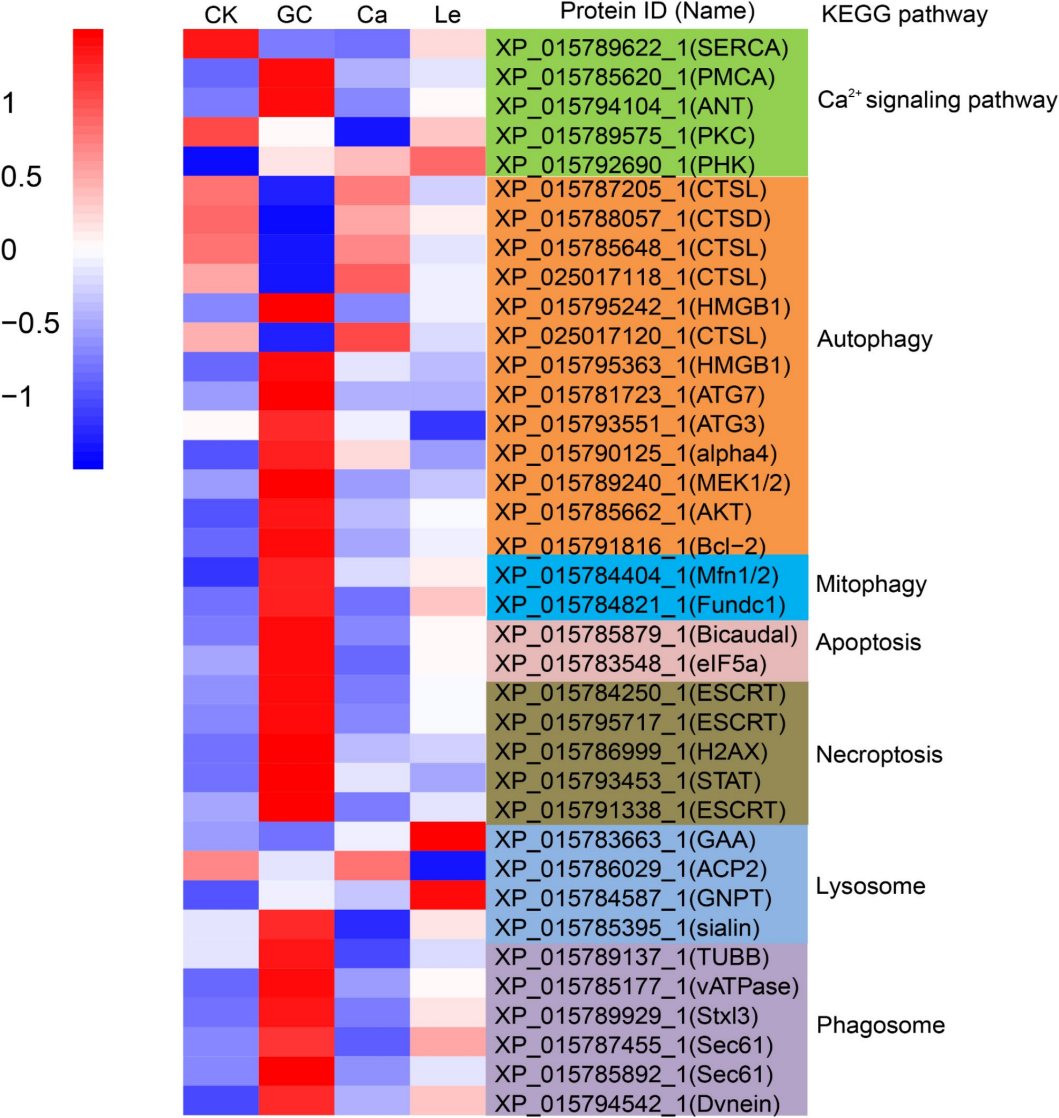
Heatmap of the differentially expressed proteins (DEPs) enriched in key KEGG pathways. Pathways include Calcium signaling pathway, Autophagy, Mitophagy, Apoptosis, Necroptosis, Phagosome, and Lysosome. GC16: GC; Control: CK; Lecithin: Le; CaCl_2_: Ca

Proteomic analysis was performed to investigate whether GC16 treatment disrupts cellular homeostasis. The DEPs corresponding to the Ca^2+^ signaling pathway, autophagy, mitophagy, apoptosis, necroptosis, phagosomes, and lysosomes were analyzed (Fig. 4). In the autophagy and mitophagy pathways, GC16 treatment increased the expression of the ubiquitin-like modifier-activating enzyme ATG7, ubiquitin-like conjugating enzyme ATG3, and B-cell lymphoma-2. In the Ca^2+^ signaling pathway, GC16 treatment inhibited the expression of sarcoplasmic/endoplasmic reticulum-type calcium-transporting ATPase. In addition, it upregulated the expression of V-type proton ATPase subunit C and eukaryotic translation initiation factor 5 in the mites. Proteomic analysis showed that GC16 treatment upregulated the expression of most DEPs related to autophagy, mitophagy, apoptosis, and necroptosis, suggesting that this treatment disrupted cellular homeostasis by disturbing autophagy, apoptosis, and necroptosis pathways.

### GC16-induced autophagy in mites and Sf9 cells

**Fig. 5:**
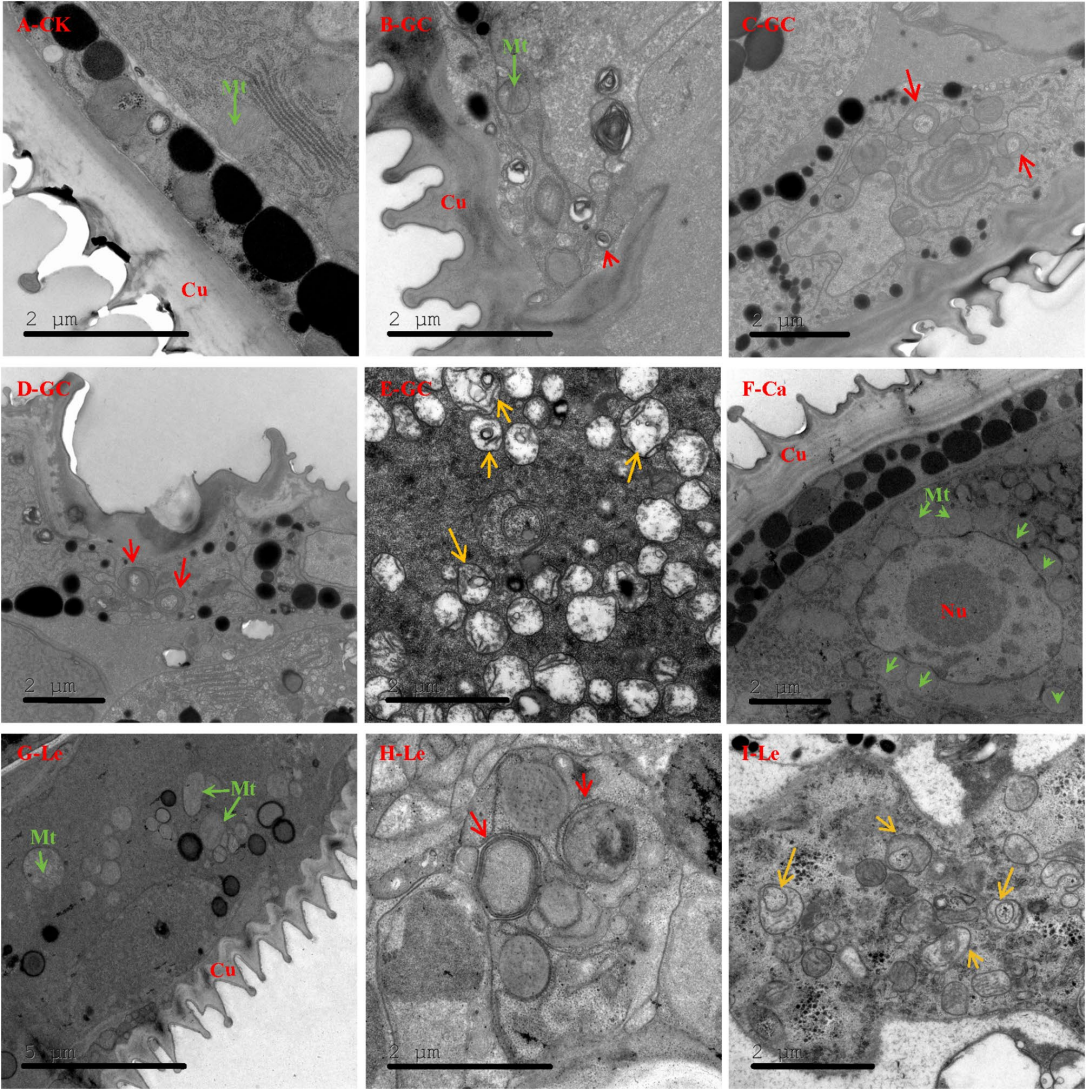
Transmission electron microscopy (TEM) was employed to investigate the induction of autophagy in *Tetranychus pueraricola*. The mites were subjected to treatments with GC16 (GC), a control group (CK), CaCl_2_ (Ca), or lecithin (Le) for a duration of 24 h. Autophagosomes and autolysosomes were delineated using red and yellow arrows, respectively. Mitochondria were identified with the label ‘Mt’ and highlighted with green arrows, while the nucleus was denoted by ‘Nu’. Panel (**A**) presents the TEM observations of *T. pueraricola* from the control (water) group. Panels (**B**-**E**) depict the TEM observations of *T. pueraricola* following GC16 treatment. Panel (**F**) illustrates the TEM observations of *T. pueraricola* in the CaCl_2_ treatment group. Panels (**G**-**I**) showcase the TEM observations of *T. pueraricola* from the lecithin treatment group

Results of transcriptomic and proteomic analyses demonstrated that GC16 affected autophagy in the mites. TEM observation was performed to further verify this phenomenon. TEM observations revealed the presence of more autophagosomes and autolysosomes in the GC16 or lecithin group than in the control group (Fig. 5), indicating that GC16 treatment triggered autophagy in mites.

**Fig. 6:**
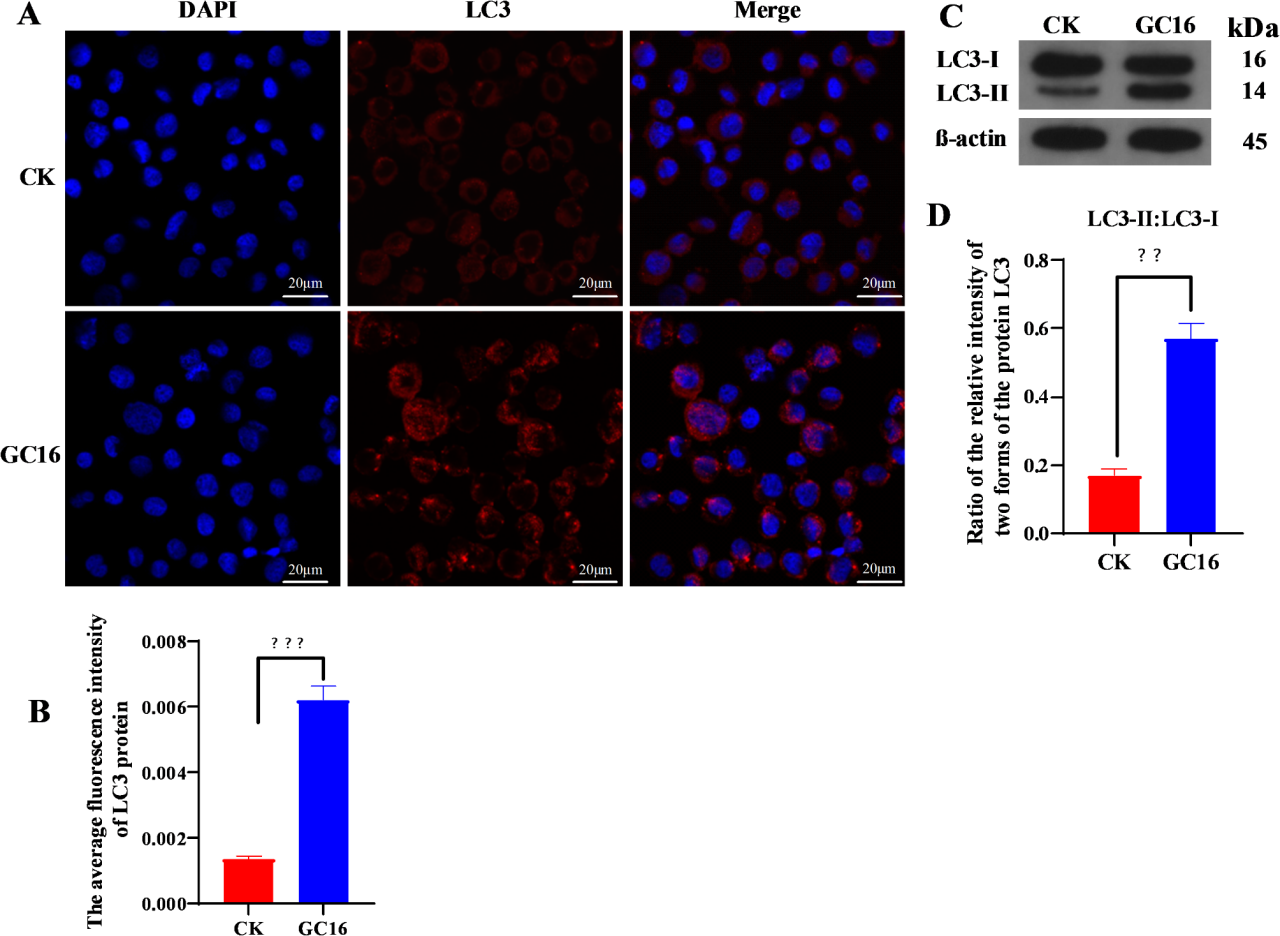
GC16 induced autophagy in Sf9 cells. (**A**) Representative fluorescence staining images of the LC3 protein in Sf9 cells treated with GC16 or water (for control, designated as CK) for 24 h (scale bar = 20 μm). The region of the LC3 protein that displays positive staining is observed to be red in color. The staining of the nucleus is visible in blue with DAPI. Autophagic vacuoles were observed to be red in color. (**B**) Average fluorescence intensity of the LC3 protein in Fig. 6A. Data are represented as mean ± SE of three replicates (*P < 0.05, **P < 0.01, and *** *P* < 0.001 with compared to control). (**C**) Western blotting analysis for expression of autophagy marker LC3 in Sf9 cells treated with GC16 for 24 h. β-Actin was used as a loading control. The blots were cropped and in supplementary file the full-length blots/gels are presented. (**D**) A quantitative analysis of western blot was shown by LC3-II/LC3-I ratios in the down panel. All data are represented as mean ± SE of three experiments in triplicate (*P < 0.05, **P < 0.01, and *** *P* < 0.001 with compared to control)

To further characterize the GC16-induced autophagy, we measured the relative concentrations of selected autophagy-related proteins using an immunofluorescence assay and western blotting. We determined the expression levels of the protein LC3 in Sf9 cells after GC16 treatment for 24 h. Based on the fluorescence images, a number of red autophagic vacuoles formation were observed clearly in the GC16 group (Fig. 6A). The fluorescence intensity of the autophagy marker LC3 was significantly stronger in the GC16-treated Sf9 cells than in the water-treated group (Fig. 6B). Moreover, Fig. 6C shows a western blot wherein two isoforms of LC3 are observed, and the conversion of LC3-I to LC3-II was regarded as an indicator of autophagosome formation. The western blot results indicated that GC16 triggered the upregulation of LC3-II (Fig. 6C) and that the ratio of LC3-II/LC3-I was increased with GC16 treatment (Fig. 6D). Overall, GC16 treatment markedly enhanced autophagosome formation in Sf9 cells in comparison to the control. These findings indicate that GC16 induces autophagy in Sf9 cells.

### GC16-induced generation of ROS in Sf9 cells

**Fig. 7:**
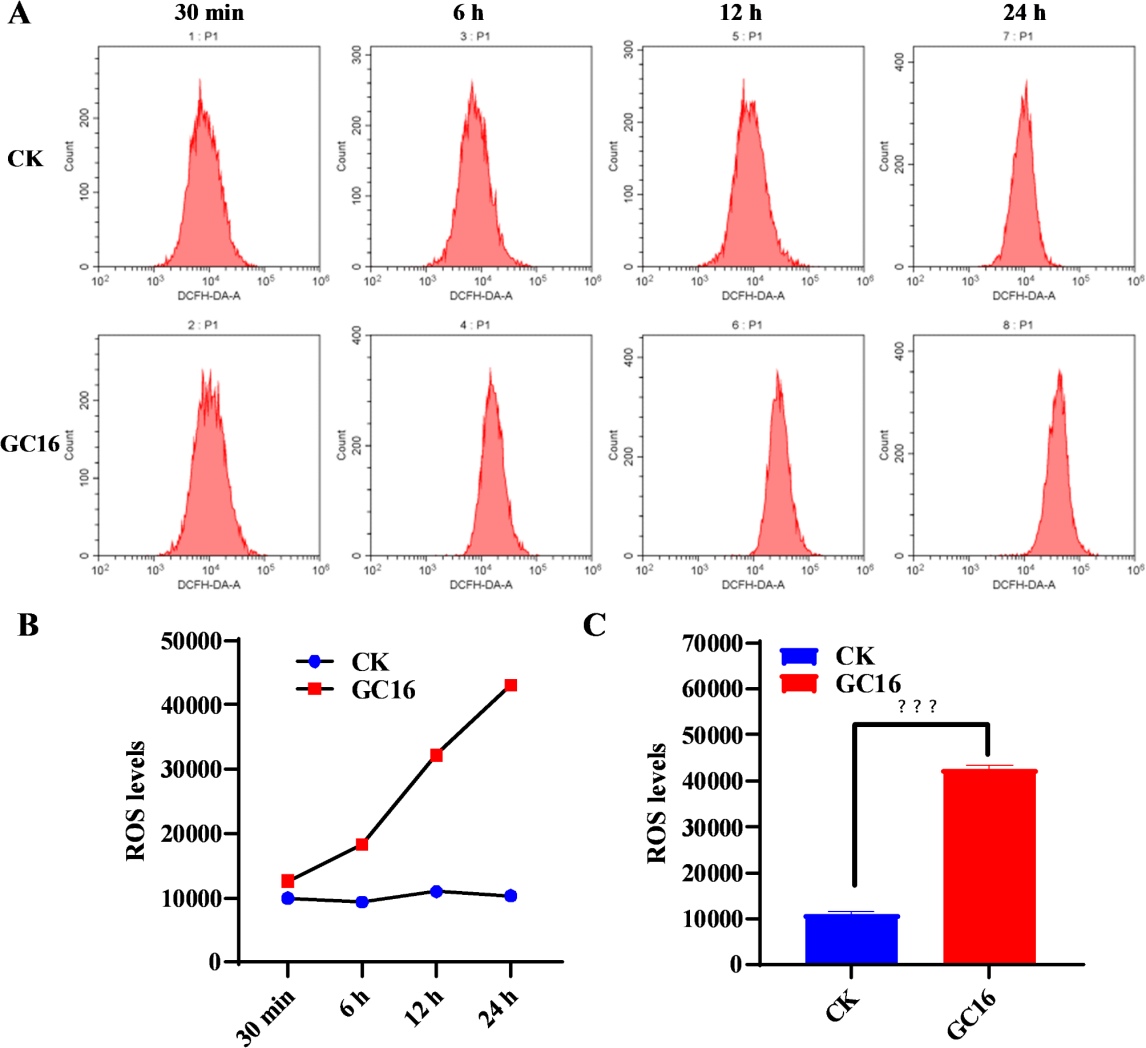
ROS levels in Sf9 cells after exposure to GC16 and control (CK). (**A**) The intracellular ROS levels tested by flow cytometry. (**B**) Numerical analysis of panel A, with the ordinate indicating the ROS level and the abscissa indicating the different processing times. (**C**) The intracellular ROS levels for 24 h

The autophagic process is frequently accompanied by alterations in ROS levels. To further investigate the changes in intracellular ROS levels during the action of GC16, we monitored ROS in Sf9 cells after continuous exposure to GC16. The cellular levels of ROS increased in Sf9 cells when exposed to GC16 for 30 min, 6, 12, and 24 h, compared to the water group (control) (Fig. 7). Moreover, the accumulation of ROS intensified with prolonged treatment duration. These results suggest that GC16 induced generation of ROS in Sf9 cells.

### GC16 induced apoptosis in Sf9 cells

**Fig. 8:**
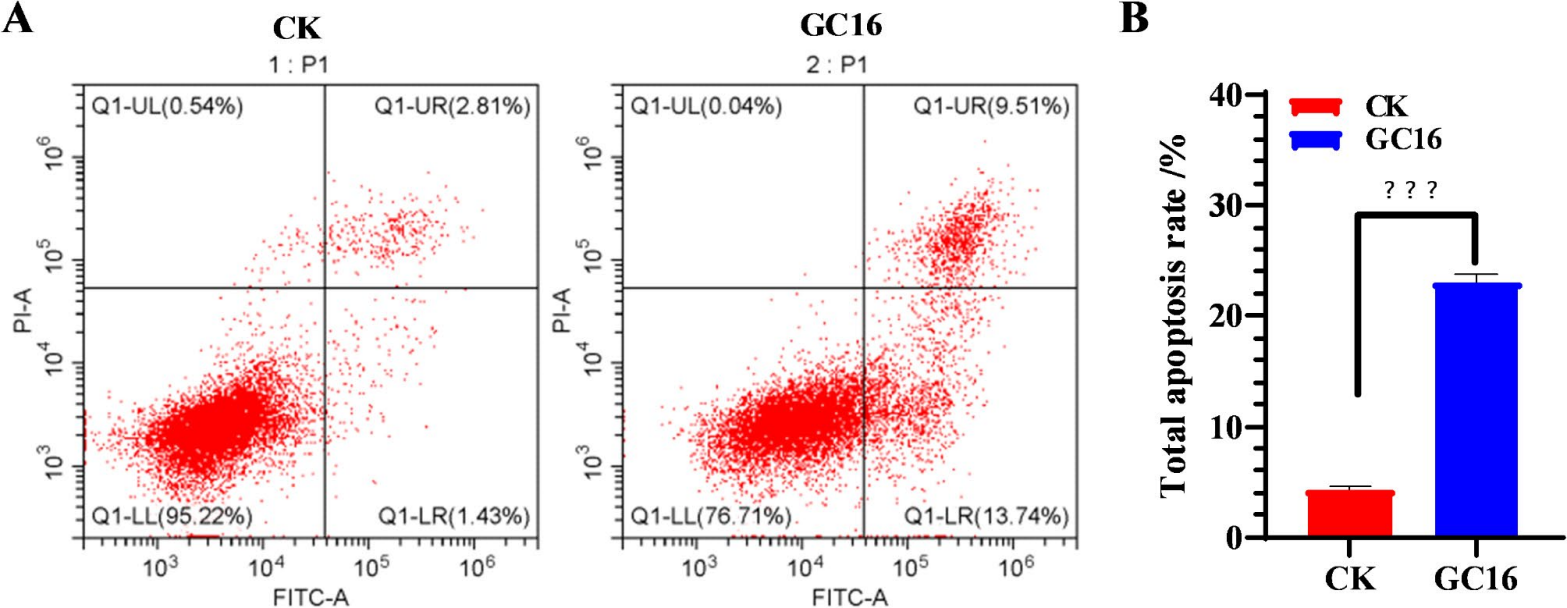
Apoptosis in Sf9 cells after exposure to GC16 (LC_50_) and control (CK). (**A**) Cell apoptosis through flow cytometry. (**B**) The total apoptosis rate in panel A was quantified

As previously demonstrated, GC16 induced cellular autophagy. To further investigate the potential impact of GC16 on apoptosis, the total apoptosis rate in Sf9 cells was quantified following treatment with GC16. Flow cytometry results demonstrated a significant increase in the total apoptosis rate of Sf9 cells exposed to GC16 for 24 h (23.01 ± 0.81%) compared with that in the water group (control) (total apoptotic rate: 4.33 ± 0.30%) (Fig. 8). These findings indicate that GC16 can induce apoptosis in insect cells.

### [Ca2+]i level

**Fig. 9:**
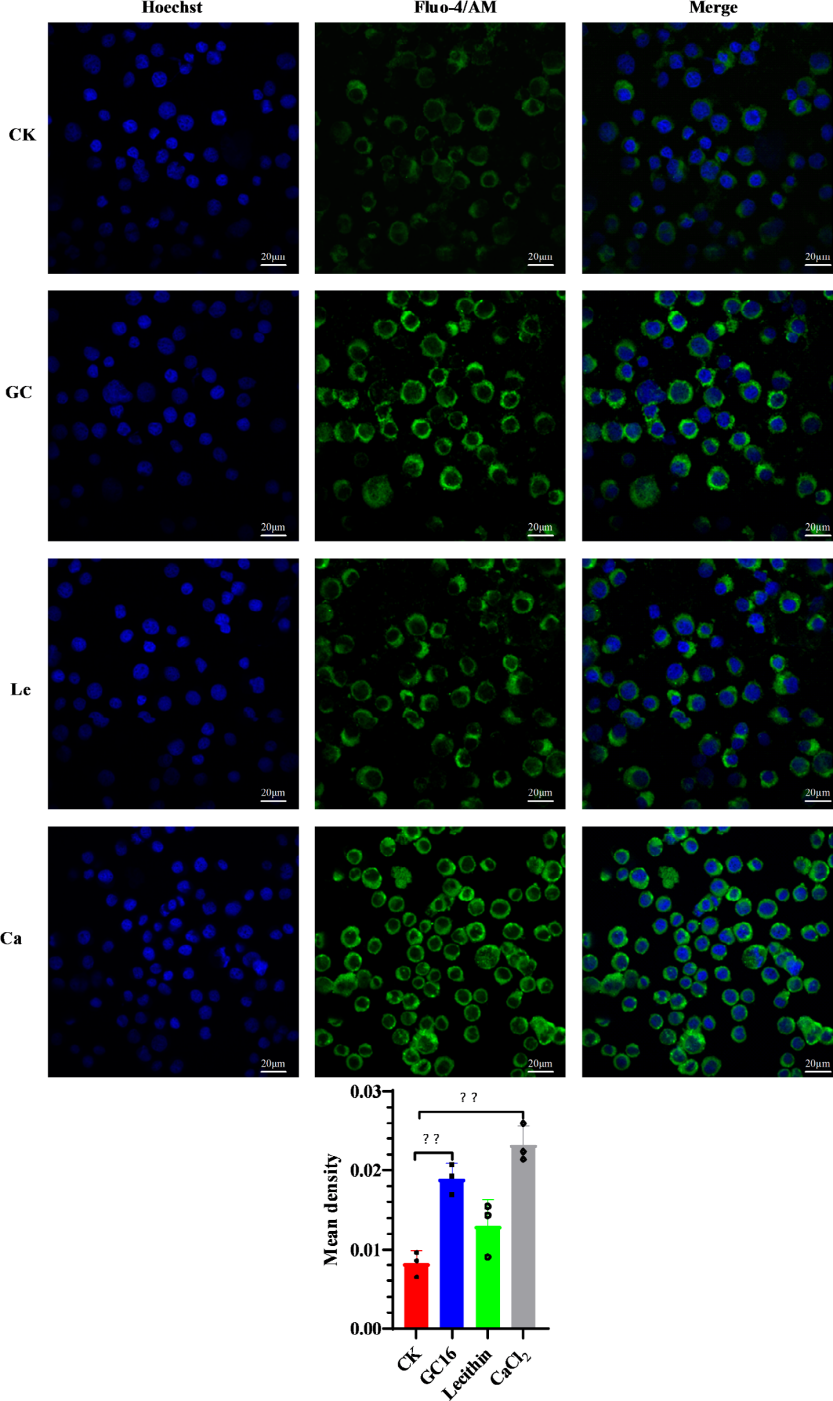
The intracellular Ca^2+^ levels in Sf9 cells. Cells were exposured to GC16, Control (CK), CaCl_2_ (Ca), and lecithin (Le), respectively. The [Ca^2+^]i level was detected by Fluo-4/AM fluorescence staining. Positively stained calcium is shown as green zones in the captured images under a microscope. Nuclei were stained with Hoechst (blue). Quantification of Ca^2+^ levels is shown in the bar chart. The bar chart indicates the mean fluorescence intensity of Fluo-4/AM in Sf9 cells. Data are shown as mean ± SE, significance was determined by *p* < 0.01 (**) as statistically different between the treatment and control according to unpaired student’s *t* test (*n* = 3)

To determine the impact of GC16 on intracellular [Ca^2+^]i levels, Fluo-4/AM staining was used to measure the concentrations of intracellular [Ca^2+^]i. [Ca^2+^]i levels were significantly higher (2.30-fold or 2.82-fold) in the GC16- or CaCl_2_-treated cells than in the control cells. These results suggested that GC16 treatment significantly increased the [Ca^2+^]i levels in the insect Sf9 cells via CaCl_2_ (Fig. 9).

## Discussion

The exploration of novel targets and mechanisms of pesticide action is challenging but crucial for supporting the development of efficient and sustainable pest control strategies [31, 49]. In the present study, we investigated the acaricidal mechanisms of GC16 by using transcriptomic and proteomic analyses to focus on the KEGG enrichment of DEGs and DEPs. We also determined the roles of calcium signaling and autophagy in the mode of action of this pesticide through fluorescent staining assay, TEM, and western blot.

GO and KEGG enrichment analyses of the transcriptome indicated that GC16 treatment affected the biogenesis and structure of the ribosomes. Ribosomes synthesize proteins from mRNA and affect cell proliferation, differentiation, and apoptosis [50]. Ribosome biogenesis creates ribosomes that are closely linked to protein synthesis, cell growth, and cell proliferation [51]. Oxidative phosphorylation is a metabolic process that occurs within the mitochondria and is responsible for generating the majority of the ATP necessary to support life [52–53]. In the present study, results suggested that GC16 treatment affected the oxidative phosphorylation pathway in mites via lecithin. This finding agrees with a previous report that this pesticide induces mitochondrial abnormalities in mites via lecithin [6]. The mitochondria play key roles in energy metabolism, apoptosis, autophagy, and innate immunity. Mitochondrial metabolites, such as ATP, ROS, and Ca^2+^, can modulate these processes [53]. Impairments in these processes may correlate with diseases such as neurodegeneration and cancer [54].

KEGG enrichment analysis of proteomics indicated that GC16 affected the calcium signaling pathway through CaCl_2_. The Ca^2+^ signaling pathway plays a role in insecticidal efficacy. The insecticidal properties of wilforine on *Mythimna separata* and the acaricidal efficacy of scopoletin against *T. cinnabarinus* have established a link to this pathway [14, 55]. To further determine the role of Ca^2+^ signaling in the mode of action of GC16, we examined the effect of GC16 treatment on [Ca^2+^]i levels. GC16 treatment significantly increased [Ca^2+^]i levels through CaCl_2_, demonstrating that the acaricidal mechanism of GC16 involved intracellular Ca^2+^ overload. In a previous study, mites became immobile and subsequently died after GC16 treatment, thus displaying symptoms of neurotoxicity [6]. Combined with those of previous studies, the results of the present study suggest that GC16 acts as a nerve agent that disrupts Ca^2+^ homeostasis. The addition of Ca^2+^ to lecithin significantly enhances its acaricidal activity [6], which is supported by another study indicating that Ca^2+^ positively affects the insecticidal efficacy of scopoletin [28]. The mechanism underlying the acaricidal activity of scopoletin involves the activation of the Orai1 Ca^2+^ channel, resulting in calcium overload due to the downregulation of the transcription factor SoxNeuroA [31]. In the presence of serine, lecithin (also called phosphatidylcholine, PC) produces phosphatidylserine (PS) via phospholipase D in vitro [56] and PS synthetase-1 in vivo [57]. PS often serves as an eat-me signal on apoptotic cell outer surfaces, attracting engulfing cells and initiating engulfment. High levels of cytoplasmic Ca^2+^ promote PS exposure, triggering engulfment [58]. Treatment with GC16, a mixture of lecithin and CaCl_2_, leads to CaCl_2_ inducing higher cellular Ca^2+^ levels, whereas PC generates more PS. Elevated Ca^2^ 

## Conclusions

We established a transcriptomic and proteomic database for *T. pueraricola* and explored the acaricidal mechanism of GC16. KEGG enrichment of transcriptomic and proteomic data consistently showed that the DEGs and DEPs in the comparison between the GC16 and control groups were enriched in the autophagy pathway. Furthermore, TEM, immunofluorescence assay, and western blot results proved that GC16 treatment triggered autophagy. Moreover, GC16 treatment increased the levels of ROS and apoptosis. Additionally, GC16 treatment significantly induced Ca^2+^ signaling, indicating the involvement of Ca^2+^ in the acaricidal mechanism of GC16. Thus, the acaricidal mechanism of GC16 involved the activation of autophagy, which may be induced by ROS generated by elevated intracellular Ca^2+^, ultimately resulting in apoptosis. This work may serve as a basis for further studying *T. pueraricola* and using GC16 to control spider mites.

## Electronic supplementary material

Below is the link to the electronic supplementary material.

Supplementary Material 1

Supplementary Material 2

## Data Availability

The datasets generated and/or analysed during the current study are available in the NCBI Sequence Read Archive (https://submit.ncbi.nlm.nih.gov/subs/sra/) under the accession number of PRJNA1049884 for transcriptomic raw data. Proteomic raw data are available in iPoX (https://www.iprox.cn/) under the accession number IPX0007704000.

## Abbreviations

TEM: Transmission electron microscopy
CAP: Chlorantraniliprole
DEGs: Differentially expressed genes
DEPs: Differentially expressed proteins
FPKM: Fragments per kilobase of transcript per million mapped reads
GO: Gene Ontology
KEGG: Kyoto Encyclopedia of Genes and Genomes
PAGE: Polyacrylamide gel electrophoresis
PB: Phosphate buffer
PCA: Principal component analysis
ROS: Reactive oxygen species
PC: Phosphatidylcholine
PS: Phosphatidylserine
