# Supplementary material for: Autophagy is involved in the toxicity of the biocontrol agent GC16 against Tetranychus pueraricola (Acari: Tetranychidae) based on transcriptomic and proteomic analyses
Source: BMC Genomics. 2025 Feb 7;26:119. doi: 10.1186/s12864-025-11312-7 (PMC11806590; doi:10.1186/s12864-025-11312-7)
Supplement: Supplementary file 1 — Supplementary Material 1 [file 12864_2025_11312_MOESM1_ESM.docx]

Supplementary file

**Table S1.** Summary of the quality of the sample transcriptomic sequencing data.

**Table S2.** The numbers of the differentially expressed genes (DEGs).

**Table S3.** The overall number of peptides and proteins identified.

**Table S4.** The numbers of the differentially expressed proteins (DEPs) and jointly expressed proteins.

**Table S5.** The list of gene ID corresponding to differentially expressed protein ID.

**Fig. S1.** Principal component analysis (PCA) of the experimental grouping of the samples for transcriptomic data. Note: The abscissa PC1 and ordinate PC2 represent the scores of the first and second ranked principal components, respectively, and the scatter color indicates the experimental grouping of the samples. GC16: GC; Control: CK; Lecithin: Le; CaCl_2_: Ca.

**Fig. S2.** Heatmap of gene expression clustering of the samples for transcriptomic data. Note: The abscissa is the sample name and the ordinate is the value normalized by gene expression FPKM. GC16: GC; Control: CK; Lecithin: Le; CaCl_2_: Ca.

**Fig. S3.** GO enrichment of the differentially expressed genes (DEGs) between GC16 (GC) and Control (CK), Lecithin (Le) and Control (CK), and CaCl_2_ (Ca) and Control (CK). The abscissa is the compared group of differential treatments, and the ordinate is GO Term.

**Fig. S4.** Principal component analysis (PCA) of the experimental grouping of the samples for proteomic data. Note: The abscissa PC1 and ordinate PC2 represent the scores of the first and second ranked principal components, respectively, and the scatter color indicates the experimental grouping of the samples. GC16: GC; Control: CK; Lecithin: Le; CaCl_2_: Ca.

**Fig. S5.** Heatmap of protein expression clustering of the samples for proteomic data. Note: The abscissa is the sample name and the ordinate is the normalized protein relative content value. GC16: GC; Control: CK; Lecithin: Le; CaCl_2_: Ca.

**Fig. S6.** GO enrichment of the differentially expressed proteins (DEPs) between GC16 (GC) and Control (CK), Lecithin (Le) and Control (CK), and CaCl_2_ (Ca) and Control (CK). The abscissa is the compared group of differential treatments, and the ordinate is GO Term.

Tables

Table S1. Summary of the quality of the sample transcriptomic sequencing data

| Sample | Library | Raw_reads | Raw_bases | Clean_reads | Clean_bases | Error_rate | Q20 | Q30 | GC_pct |
| --- | --- | --- | --- | --- | --- | --- | --- | --- | --- |
| Ca1 | FRAS230299520-1r | 42187170 | 6.33G | 41351934 | 6.2G | 0.03 | 97.94 | 93.81 | 39.19 |
| Ca2 | FRAS230299521-1r | 42412040 | 6.36G | 41602958 | 6.24G | 0.03 | 97.96 | 93.81 | 37.78 |
| Ca3 | FRAS230299522-1r | 42248254 | 6.34G | 41576666 | 6.24G | 0.03 | 97.72 | 93.2 | 37.11 |
| CK1 | FRAS230299517-1r | 41969354 | 6.3G | 41078762 | 6.16G | 0.03 | 97.75 | 93.27 | 37.69 |
| CK2 | FRAS230299518-1r | 41968618 | 6.3G | 41160708 | 6.17G | 0.03 | 97.88 | 93.6 | 37.81 |
| CK3 | FRAS230299519-1r | 41669778 | 6.25G | 40825532 | 6.12G | 0.03 | 97.87 | 93.59 | 38.49 |
| GC1 | FRAS230299513-1r | 42021064 | 6.3G | 41191610 | 6.18G | 0.03 | 97.88 | 93.64 | 38.32 |
| GC2 | FRAS230085868-2r | 42911032 | 6.44G | 41960262 | 6.29G | 0.03 | 97.86 | 93.56 | 37.71 |
| GC3 | FRAS230299514-1r | 44392352 | 6.66G | 42694520 | 6.4G | 0.03 | 97.66 | 93.11 | 37.58 |
| Le1 | FRAS230299515-1r | 42136416 | 6.32G | 41437022 | 6.22G | 0.03 | 97.87 | 93.63 | 37.85 |
| Le2 | FRAS230299516-1r | 42386014 | 6.36G | 41806006 | 6.27G | 0.03 | 97.77 | 93.37 | 37.31 |
| Le3 | FRAS230085870-1r | 42010564 | 6.3G | 40999958 | 6.15G | 0.03 | 97.88 | 93.63 | 37.58 |

Table S2. The numbers of the differentially expressed genes (DEGs)

| Compare | All | Up | Down | Threshold |
| --- | --- | --- | --- | --- |
| GC.vs.CK | 2717 | 1338 | 1379 | DESeq2 pvalue<=0.05 \|log_2_FoldChange\|>=0.0 |
| Le.vs.CK | 2212 | 1060 | 1152 | DESeq2 pvalue<=0.05 \|log_2_FoldChange\|>=0.0 |
| Ca.vs.CK | 1729 | 811 | 918 | DESeq2 pvalue<=0.05 \|log_2_FoldChange\|>=0.0 |

Table S3. The overall number of peptides and proteins identified

| Name | Total spectra | Matched spectrum | Peptide | Identified protein |
| --- | --- | --- | --- | --- |
| ALL | 598711 | 498050 | 29212 | 3277 |

Table S4. The numbers of the differentially expressed proteins (DEPs) and jointly expressed proteins

| Compared Samples | Num. of Total Quant. | Regulated  Type | FC>1.2 | FC>1.3 | FC>1.5 | FC>2.0 |
| --- | --- | --- | --- | --- | --- | --- |
| GC.vs.CK | 2882 | up | 506 | 446 | 338 | 188 |
|  |  | down | 95 | 65 | 36 | 16 |
| Le.vs.CK | 2867 | up | 216 | 168 | 105 | 43 |
|  |  | down | 34 | 24 | 15 | 6 |
| Ca.vs.CK | 2863 | up | 80 | 61 | 36 | 19 |
|  |  | down | 50 | 39 | 26 | 13 |

Note: Compared Samples: compared sample pairs, the former than the latter. Num. of Total Quant.: jointly identified proteins in the two groups of samples. FC (fold-change): the fold difference threshold. Screening criteria was set at Pvalue≤0.05.

Table S5. The list of gene ID corresponding to differentially expressed protein ID

| Protein ID | Gene ID |
| --- | --- |
| XP_015789622_1(SERCA) | tetur18g02100 |
| XP_015785620_1(PMCA) | tetur09g02350 |
| XP_015794104_1(ANT) | tetur130g00030 |
| XP_015789575_1(PKC) | tetur18g02560 |
| XP_015792690_1(PHK) | tetur31g00500 |
| XP_015787205_1(CTSL) | tetur12g01860 |
| XP_015788057_1(CTSD) | tetur14g03010 |
| XP_015785648_1(CTSL) | tetur09g04400 |
| XP_025017118_1(CTSL) | tetur12g01820 |
| XP_015795242_1(HMGB1) | tetur02g08340 |
| XP_025017120_1(CTSL) | tetur12g01830 |
| XP_015795363_1(HMGB1) | tetur02g08260 |
| XP_015781723_1(ATG7) | tetur04g02140 |
| XP_015793551_1(ATG3) | tetur37g00950 |
| XP_015790125_1(alpha4) | tetur20g01120 |
| XP_015789240_1(MEK1/2) | tetur17g02600 |
| XP_015785662_1(AKT) | tetur01g00320 |
| XP_015791816_1(Bcl-2) | tetur27g00030 |
| XP_015784404_1(Mfn1/2) | tetur01g10570 |
| XP_015784821_1(Fundc1) | tetur08g00970 |
| XP_015785879_1(Bicaudal) | tetur09g02210 |
| XP_015783548_1(eIF5a) | tetur01g12280 |
| XP_015784250_1(ESCRT-Ⅲ) | tetur07g08047 |
| XP_015795717_1(ESCRT-Ⅲ) | tetur03g06050 |
| XP_015786999_1(H2AX) | tetur11g02430 |
| XP_015793453_1(STAT) | tetur36g00600 |
| XP_015791338_1(ESCRT-Ⅲ) | tetur25g01140 |
| XP_015783663_1(GAA) | tetur06g02700 |
| XP_015786029_1(ACP2) | tetur01g10000 |
| XP_015784587_1(GNPT) | tetur07g06510 |
| XP_015785395_1(sialin) | tetur08g06870 |
| XP_015789137_1(TUBB) | tetur17g04312 |
| XP_015785177_1(vATPase) | tetur08g03920 |
| XP_015789929_1(Stxl3) | tetur19g00580 |
| XP_015787455_1(Sec61) | tetur12g04280 |
| XP_015785892_1(Sec61) | tetur09g02970 |
| XP_015794542_1(Dvnein) | tetur02g12820 |


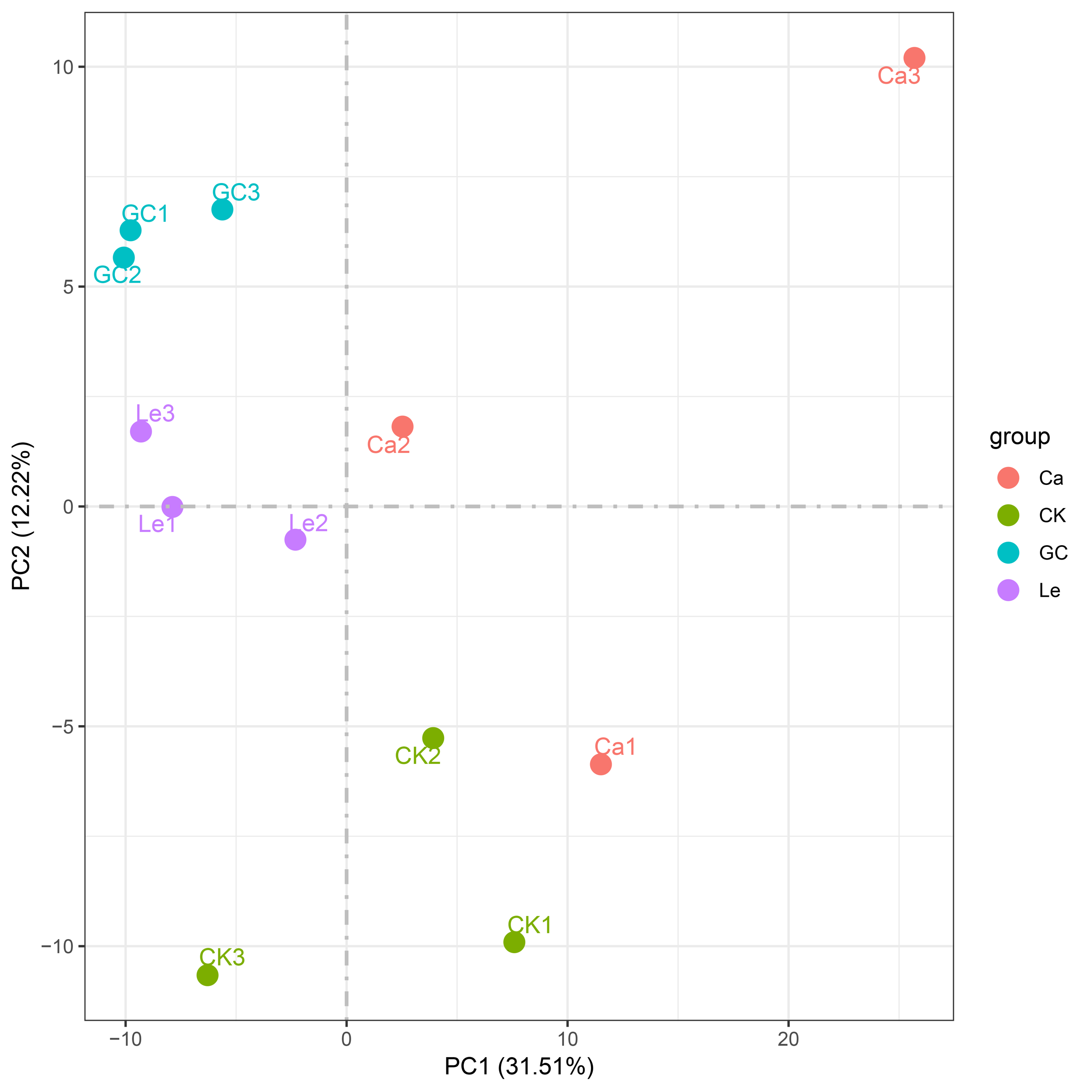


Figure S1. Principal component analysis (PCA) of the experimental grouping of the samples for transcriptomic data. Note: The abscissa PC1 and ordinate PC2 represent the scores of the first and second ranked principal components, respectively, and the scatter color indicates the experimental grouping of the samples. GC16: GC; Control: CK; Lecithin: Le; CaCl_2_: Ca.


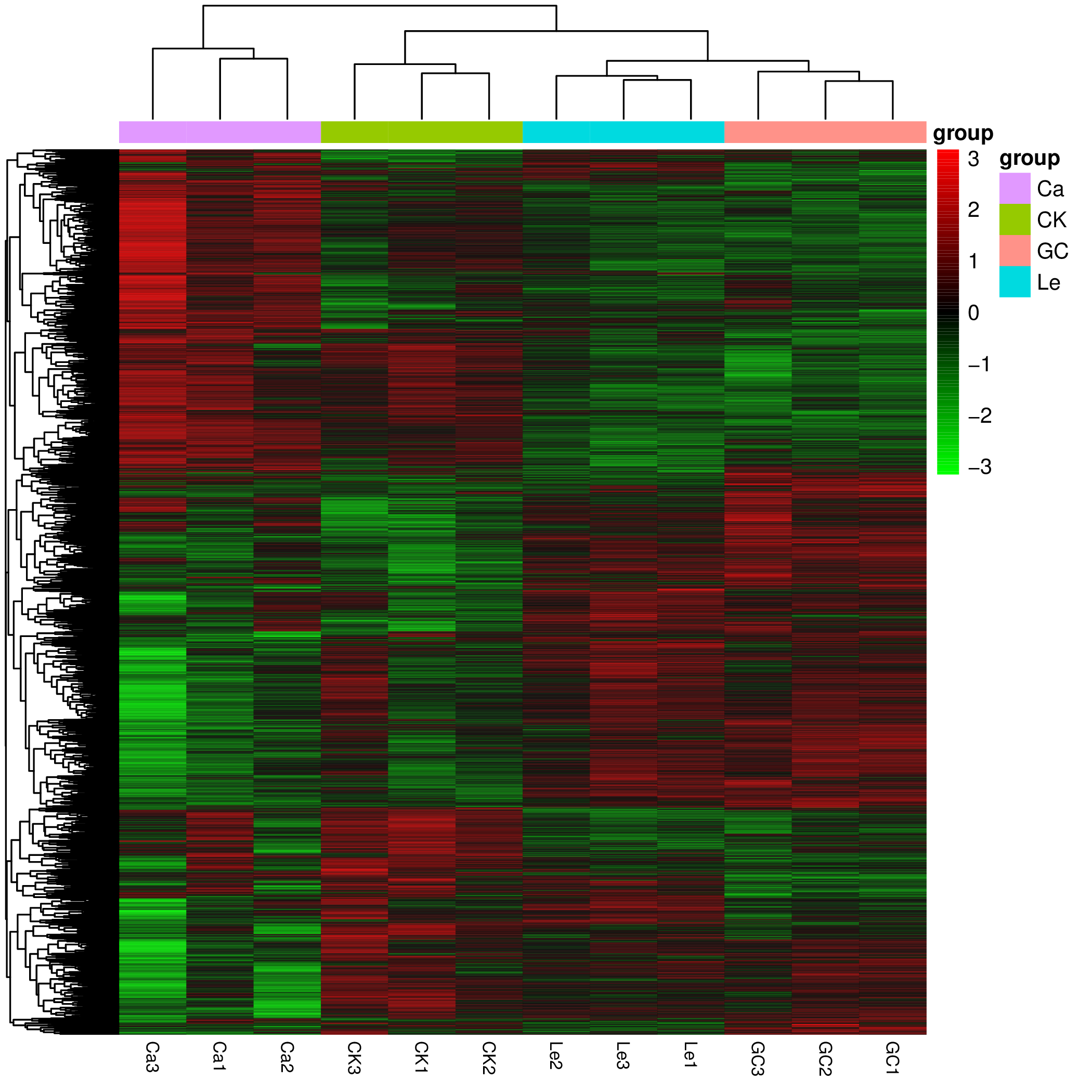


Figure S2. Heatmap of gene expression clustering of the samples for transcriptomic data. Note: The abscissa is the sample name and the ordinate is the value normalized by gene expression FPKM. GC16: GC; Control: CK; Lecithin: Le; CaCl_2_: Ca.


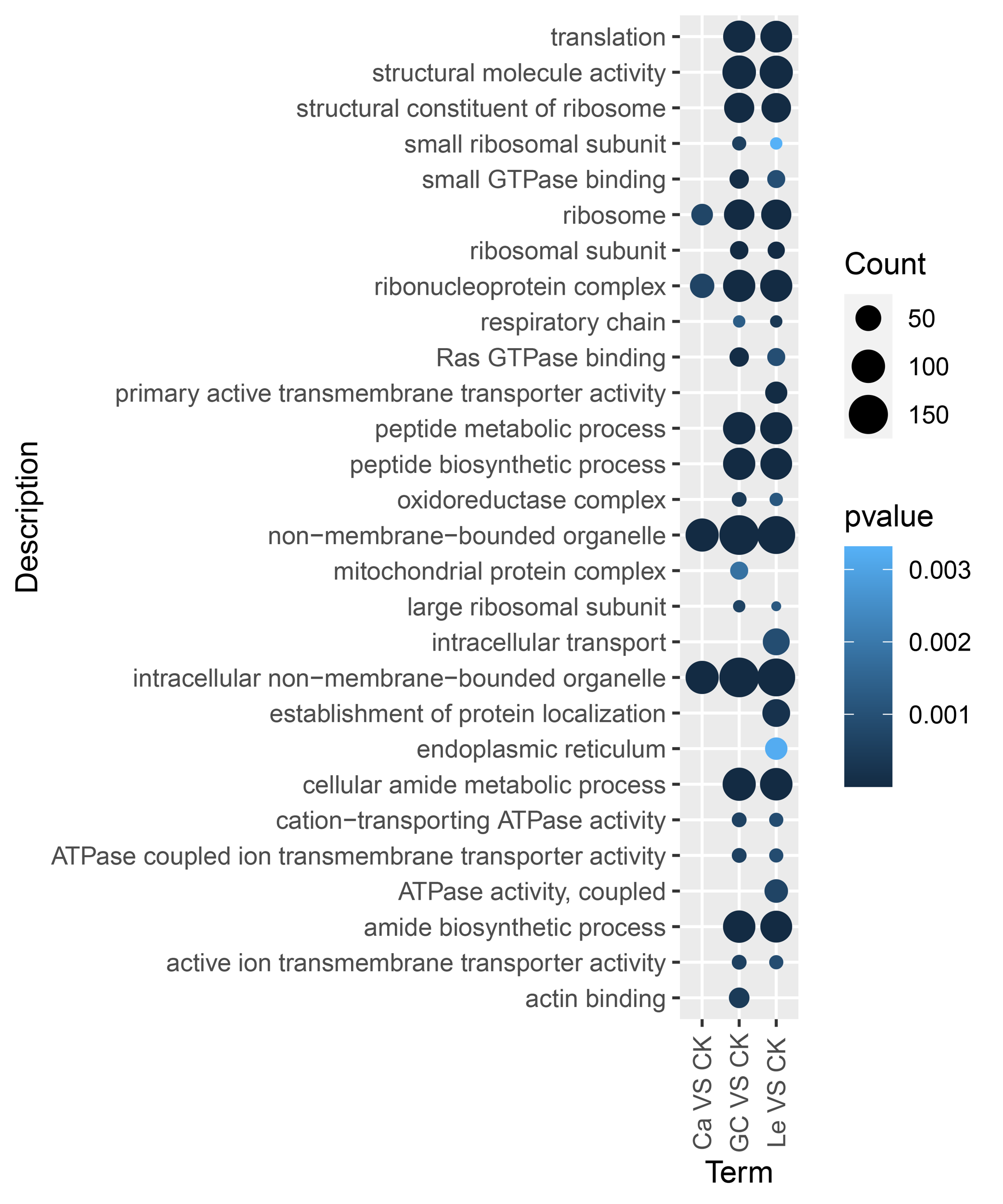


Figure S3. GO enrichment of the differentially expressed genes (DEGs) between GC16 (GC) and Control (CK), Lecithin (Le) and Control (CK), and CaCl_2_ (Ca) and Control (CK). The abscissa is the compared group of differential treatments, and the ordinate is GO Term.


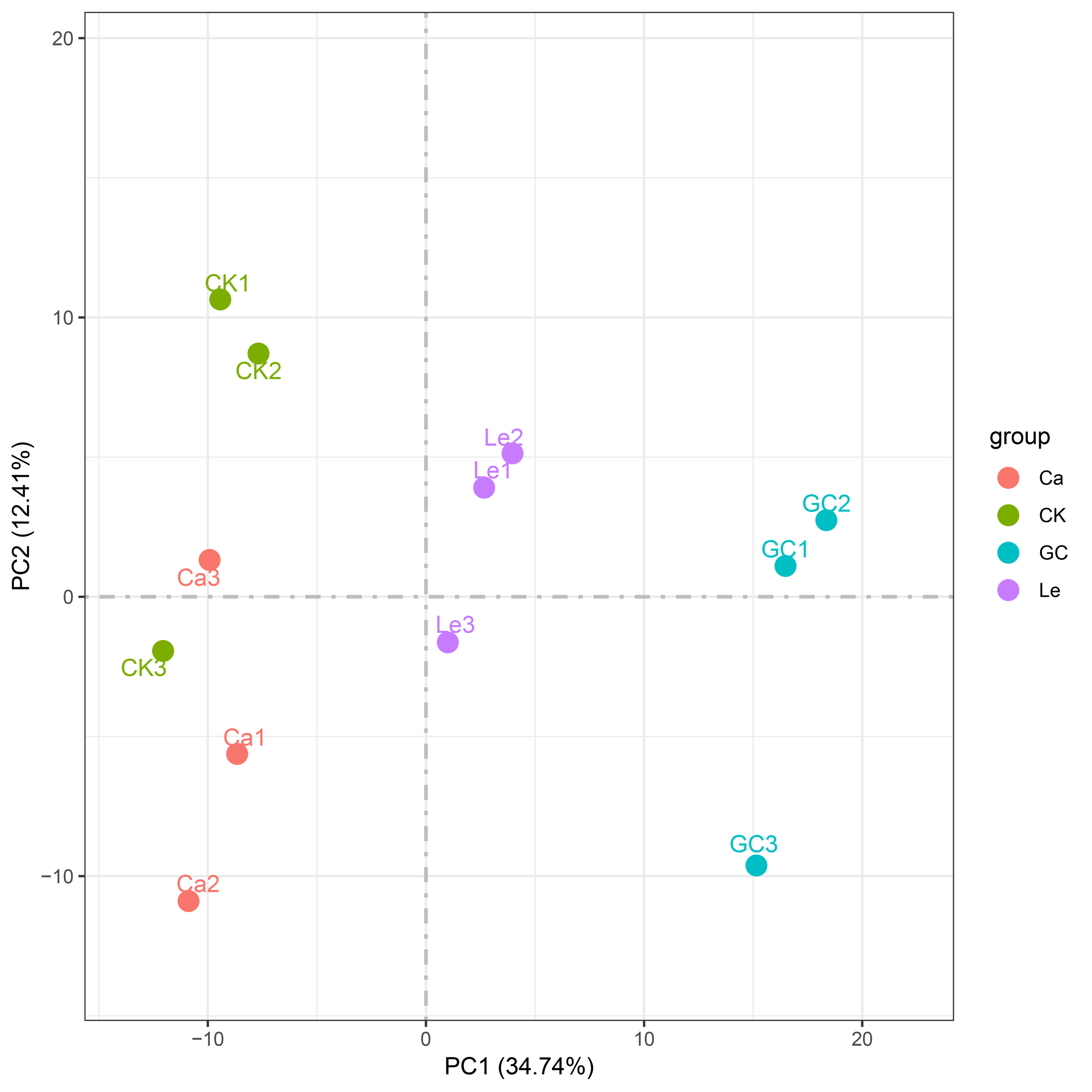


Figure S4. Principal component analysis (PCA) of the experimental grouping of the samples for proteomic data. Note: The abscissa PC1 and ordinate PC2 represent the scores of the first and second ranked principal components, respectively, and the scatter color indicates the experimental grouping of the samples. GC16: GC; Control: CK; Lecithin: Le; CaCl_2_: Ca.


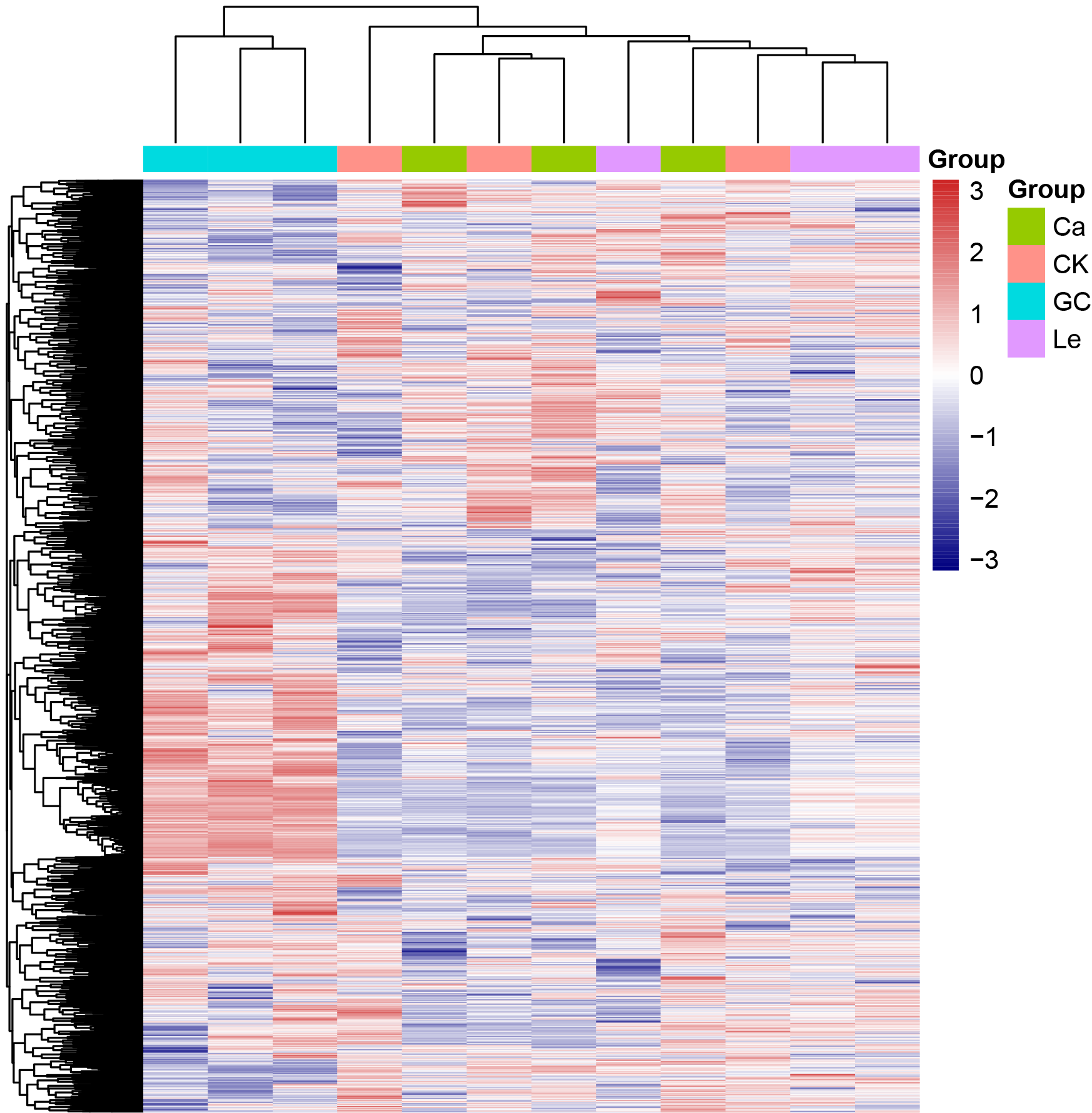


Figure S5. Heatmap of protein expression clustering of the samples for proteomic data. Note: The abscissa is the sample name and the ordinate is the normalized protein relative content value. GC16: GC; Control: CK; Lecithin: Le; CaCl_2_: Ca.


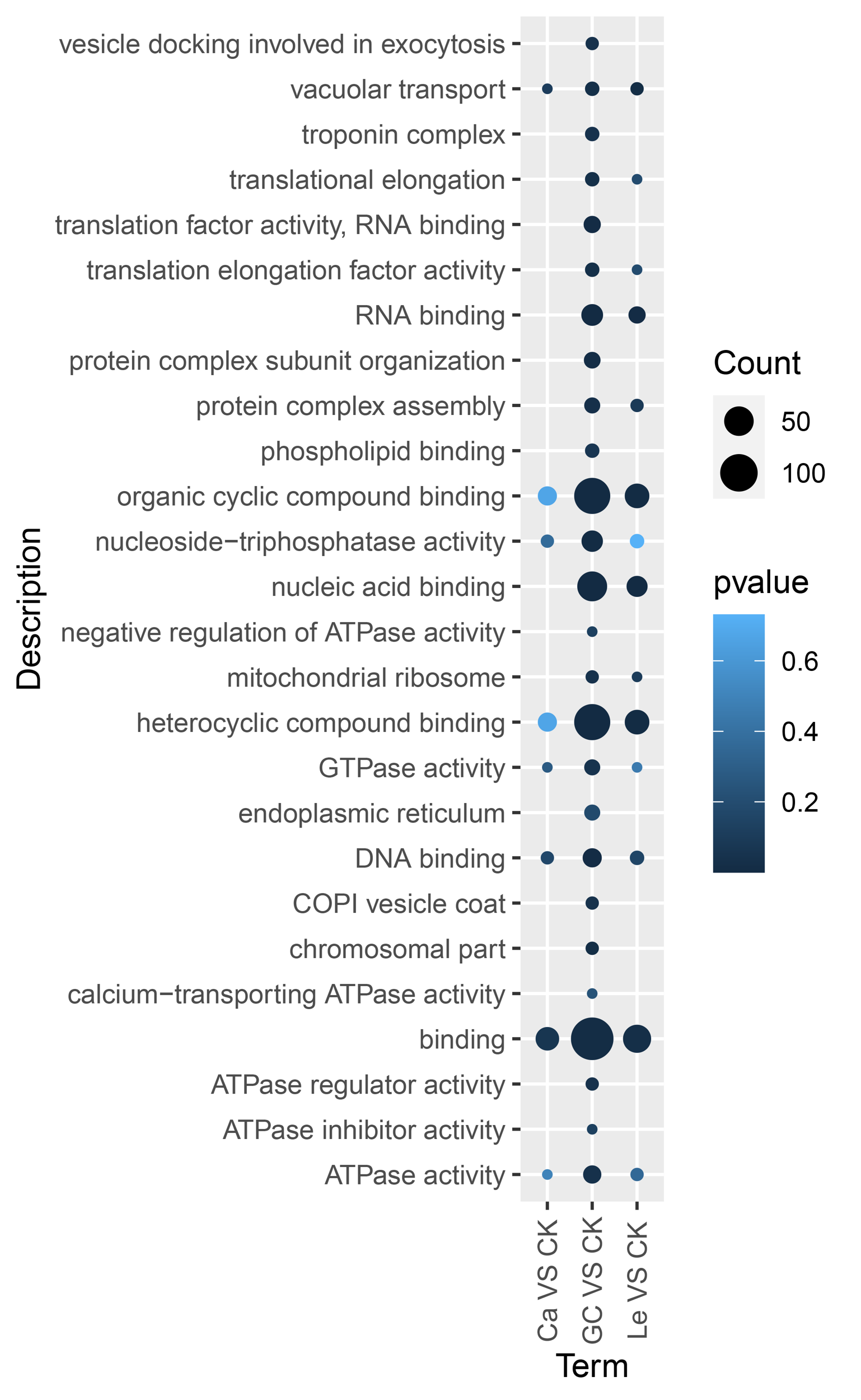


Figure S6. GO enrichment of the differentially expressed proteins (DEPs) between GC16 (GC) and Control (CK), Lecithin (Le) and Control (CK), and CaCl_2_ (Ca) and Control (CK). The abscissa is the compared group of differential treatments, and the ordinate is GO Term.
