# Supplementary material for: Autophagy is involved in the toxicity of the biocontrol agent GC16 against Tetranychus pueraricola (Acari: Tetranychidae) based on transcriptomic and proteomic analyses
Source: BMC Genomics. 2025 Feb 7;26:119. doi: 10.1186/s12864-025-11312-7 (PMC11806590; doi:10.1186/s12864-025-11312-7)
Supplement: Supplementary file 2 — Supplementary Material 2 [file 12864_2025_11312_MOESM2_ESM.docx]

Figure A1. The original, whole gels and blots images for Figure 6. Western blotting analysis for expression of autophagy marker LC3 in Sf9 cells treated with GC16 or CK. β-actin was used as a loading control .
